# Aquaporin-1 Translocation and Degradation Mediates the Water Transportation Mechanism of Acetazolamide

**DOI:** 10.1371/journal.pone.0045976

**Published:** 2012-09-21

**Authors:** Jianzhao Zhang, Yu An, Junwei Gao, Jing Han, Xueyang Pan, Yan Pan, Lu Tie, Xuejun Li

**Affiliations:** 1 State Key Laboratory of Natural and Biomimetic Drugs, Department of Pharmacology, School of Basic Medical Sciences, Peking University, Beijing, China; 2 Institute of Systems Biomedicine, Peking University, Beijing, China; Universidade Federal do Rio de Janeiro, Brazil

## Abstract

**Background:**

Diuretic agents are widely used on the treatment of water retention related diseases, among which acetazolamide (AZA) acts originally as a carbonic anhydrase (CA) inhibitor. Aquaporin-1 (AQP1) being located in renal proximal tubules is required for urine concentration. Previously our lab has reported AZA putatively modulated AQP1. Aim of this study is to testify our hypothesis that regulating AQP1 may mediate diuretic effect of AZA.

**Methodology/Principal Findings:**

For *in vivo* study, we utilized Sprague Dawley rats, as well as AQP1 knock-out (AQP1^−/−^) mice to examine urine volume, and human kidney-2 (HK-2) cell line was used for *in vitro* mechanism study. In our present study we found that AZA decreased CAs activity initially but the activity gradually recovered. Contrarily, diuretic effect was consistently significant. AQP1 protein expression was significantly decreased on day 7 and 14. By utilizing AQP1^−/−^ mice, we found diuretic effect of AZA was cancelled on day 14, while urine volume continuously increased in wild-type mice. Surface plasmon resonance (SPR) results indicated AQP1 was physiologically bound by myosin heavy chain (MHC), immunoprecipitation and immunofluorescence results confirmed this protein interaction. *In vitro* study results proved AZA facilitated AQP1 translocation onto cell membrane by promoting interaction with MHC, dependent on ERK/ myosin light chain kinase (MLCK) pathway activation. MHC inhibitor BDM and ERK inhibitor U0126 both abolished above effect of AZA. Eventually AZA induced AQP1 ubiquitination, while proteasome inhibitor MG132 reversed AZA's down-regulating effect upon AQP1.

**Conclusions/Significance:**

Our results identified AZA exerted diuretic effect through an innovative mechanism by regulating AQP1 and verified its inhibitory mechanism was via promoting MHC-dependent translocation onto cell membrane and then ubiquitin mediated degradation, implicating a novel mechanism and target for diuretic agent discovering.

## Introduction

Aquaporin-1 (AQP1) was the first water channel to be identified [1] among 13 types of mammalian aquaporins (AQP 0–12) known up to now. It is widely distributed in erythrocytes, apical brush border and in basolateral membranes of proximal tubular epithelial cells and descending limb of Henle's loop, descending vasa recta endothelia and other organs [1]–[4]. The physiological and pathophysiological role of AQP1 in kidney has been well documented. Now we know it is considered to be closely related to urine concentration [5], [6], AQP1 knock-out mice displays symptom of polyuria. Consistently it has been reported that increased expression of AQP1 in kidney is involved in male spontaneously hypertensive rats [7]. Besides of water transportation function, it was also demonstrated that AQP1 facilitates both kidney proximal tubule cells [8] and tumor cells migration [9]. Thus, considering important role of AQP1 in urine concentration, down-regulating and/or inhibiting AQP1 by small molecular modulator may cause diuretic effect.

Acetazolamide (AZA) is a potent inhibitor of carbonic anhydrases (CAs), which catalyze the equilibration of carbon dioxide and carbonic acid and plays a key role in NaHCO_3_ re-absorption and acid secretion in the process of urine formation. AZA exerts its diuretic role by inhibiting both cytoplasm form CAII and membrane-bound form CAIV located in renal proximal tubular epithelial cells, which catalyze the equilibration between carbon dioxide and carbonic acid and mediate re-absorption of HCO_3_^−^. Thus, after CAs activity is inhibited by AZA, HCO_3_^−^ re-absorption is suppressed, resulting in increase of HCO_3_^−^ excretion. CAs inhibition also decreases the production of H^+^ and reduces the H^+^-Na^+^ exchange, resulting in suppression of Na^+^ re-absorption and H_2_O re-absorption in proximal tubules. Eventually CAs inhibition by AZA induces a mild diuretic effect. As a diuretic agent, AZA is clinically used to treat edema due to congestive heart failure and drug-induced water retention. However, the rapid development of tolerance has limited its application. Previous study in our lab [10], [11] and other groups [12], [13] has suggested AZA could be a potent inhibitor of AQP1. Since AQP1 is the mainly water channel expressed on the proximal tubule epithelial and is considered to have the capacity to reabsorb 90% of the glomerular filtrate[14] while this segment is the right action site for AZA, we hypothesized that the diuretic effect of AZA may be due to its capacity of affecting AQP1 besides of inhibiting CAs.

The purpose of the present study is to determine whether the diuretic effect of AZA is partially mediated by modulating AQP1. The mechanism of AQP1 reduction after administration of AZA was also discussed. Our data suggested that AZA promoted interactions between AQP1 and myosin heavy chain (MHC). Consequently more AQP1 was carried to cell membrane and then ubiquitination of AQP1 followed by the degradation through proteasome occurred. Therefore, decreasing AQP1 protein expression was proved to be a novel diuretic mechanism of AZA.

## Results

### AZA induced diuretic effect not only by inhibiting CAs activity but also by decreasing AQP1

**Figure 1:**
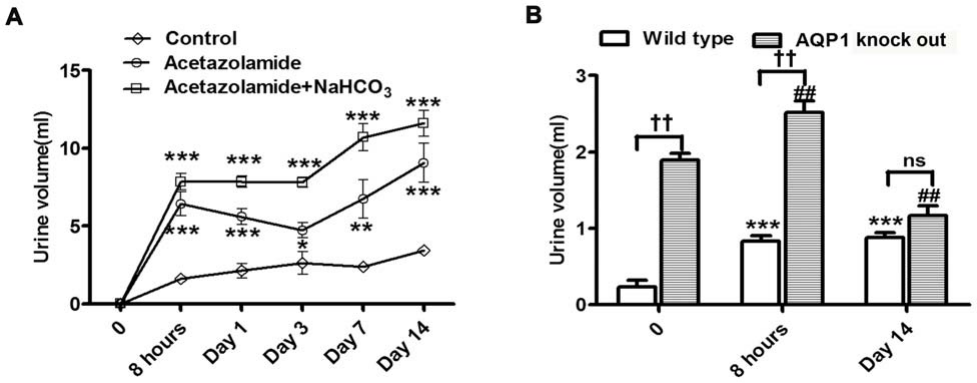
Diuretic effect of AZA on rats and knock out mice. **A**, The effect of oral administration of AZA (40 mg/kg/day) with or without NaHCO_3_ (30 mg/kg/day) on urine volume in rats. Urine outcomes were determined in three separate groups (n = 6 each) of rats (vehicle, AZA, AZA+NaHCO_3_). Urine was collected after different administration times (8 hours, 1 day, 3 days, 7 days and 14 days). Values are presented as means±S.E.M. * *p*<0.05, ** *p*<0.01, *** *p*<0.001 compared to the control group. **B**, The effect of oral administration of AZA (40 mg/kg/day) on urine volume in AQP1^−/−^ mice. Urine outcomes were determined in 2 separate groups (n = 6 each) of mice (wild mice treated with AZA, AQP1^−/−^ mice treated with AZA). Urine was collected after different administration times (before dosing, 8 hours and 14 days after dosing). Values are shown as means±S.E.M. *** *p*<0.001 compared to the wild type controls. ††*p*<0.01 compared with counterparts. ##*p*<0.01 compared to the AQP1^−/−^ controls. Ns, non-significant.

**Figure 2:**
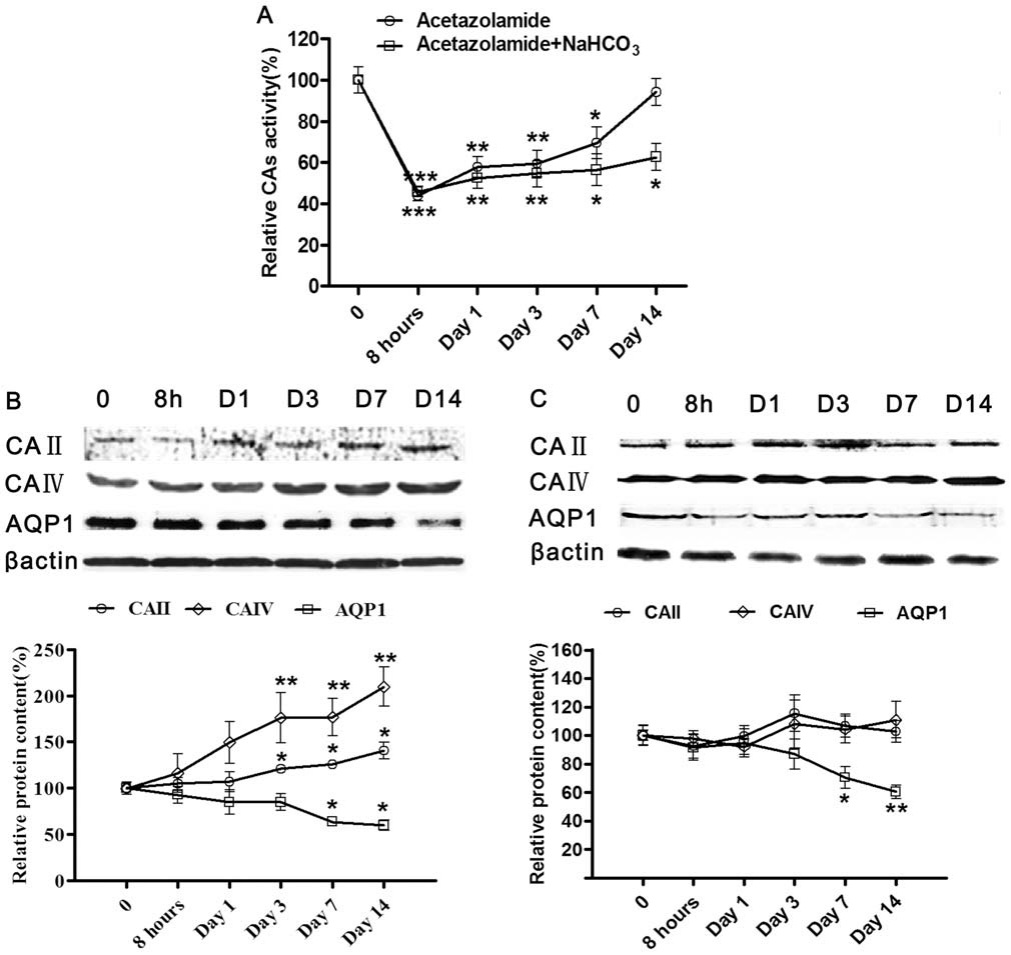
AZA induced CAs activity recovery and AQP1 reduction. **A**, The time course of total CAs activity in rat kidney cortex. The carbonic anhydrase activity was assayed by endpoint colorimetric microtechnique after the treatment with AZA (40 mg/kg/day) or AZA (40 mg/kg/day) and NaHCO_3_ (30 mg/kg/day) at the indicated time. Results are expressed as a percentage of the control. Values are the means±S.E.M. * *p*<0.05, ** *p*<0.01, *** *p*<0.001 compared to control. **B and C**, CAs and AQP1 extracted from rat kidney cortex were examined by immunoblotting in B and C. Each lane was loaded with 60 μg of total protein from rats at various times after AZA (40 mg/kg/day) (B) or combination with NaHCO_3_ (30 mg/kg/day) (C) treatment. The representative blotting images of CA II, CA IV and AQP1 are shown with β-actin as an internal control. Summary data is shown in down panels. Results are expressed as a percentage of the control. Values are the means±S.E.M. * *p*<0.05, ** *p*<0.01 compared to control.

Firstly, we found single administration of AZA caused a remarkable increase in urine volume of rats in the first 8 hours. Then urine volume was maintained on a high plateau until administration for 3 days and sustained to increase on day 7 and day 14 (Fig 1A). To examine whether AZA exerts diuretic effect only by inhibiting CAs activity, we measured CAs activity and expression meanwhile. However, on the contrary, CAs activity showed different changes from urine volume trends. Total carbonic anhydrase activity of the kidney cortex was decreased markedly within 8 hours after treated solely with AZA, but gradually recovered from day 1 and even returned to control value on day 14 (Fig 2A). We also found CAs proteins expression was induced by AZA. A 1.21-fold increment of carbonic anhydrase II protein content occurred on day 3 of treatment, which increased to 1.26-fold and 1.41-fold of the control on day 7 and 14 respectively. In the same manner, carbonic anhydrase IV protein level was increased to 1.76-fold, 1.77-fold and 2.10-fold on day 3, 7, 14, respectively compared with control. Thus single application of AZA increased carbonic anhydrases activity and expression in a time-dependent manner (Fig 2B), and theoretically this recovery means decrease of urine output, contradicting our result that urine volume was continuously increased during treatment of AZA. This contradiction indicated that a novel diuretic mechanism of AZA other than inhibiting CAs may exist.

To further testify that AZA induced diuretic effect not only by inhibiting CAs,we combined AZA with NaHCO_3_ as clinically used. NaHCO_3_ was commonly used to improve metabolic acidosis induced by AZA. The combination of AZA and NaHCO_3_ significantly reduced carbonic anhydrase activity within 8 hours as well. Moreover, in this combination condition recovery of carbonic anhydrase activity was inhibited. On day 14 of administration, CAs activity only recovered to 62.65% of control. The expression of carbonic anhydrase II and IV was not increased either (Fig 2A). Thus, without CAs activity recovery, AZA accompanied with NaHCO_3_ induced a more significant diuretic response in rats than administration solely with AZA (Fig. 1A).

Above results suggested that AZA could increase rats' urine volume lasting from 8 hours to 14 days, despite whether CAs activity and expression recovered or not, so what is CAs inhibition independent diuretic mechanism of AZA? Previous study has implicated AZA was a potential inhibitor of AQP1, which plays an important role in urine concentration. Therefore, next we examined expression of AQP1 in rats' kidneys.

Interestingly, the time course of AQP1 content was similar in AZA treated animals with or without combination with NaHCO_3_. No change in protein content occurred at time points of 8 hours, days 1, 3 post-treatment. However, a significant decrease to 63.64% of the control level was detected on the 7 day of solo AZA treatment, which decreased to 59.95% on day 14. When combined with NaHCO_3_, AZA reduced AQP1 protein expression to 70.54% and 60.3% of the control on day 7 and 14, respectively (Fig 2C). In addition, so as to exclude the impact of NaHCO_3_ only, we found single use of NaHCO_3_ produced a slight diuretic effect and did not influence CAs and AQP1 (see Fig S1 and discussion).

Furthermore, to determine the role of AQP1 in diuretic effect induced by AZA, AQP1^−/−^ mice were utilized. Fig 1B showed urine outputs of AQP1^−/−^ mice were more than that of wild mice in normal condition as previously reported [5](*P*<0.001). AZA induced diuretic effects in both AQP1^−/−^ mice and wild-type mice after 8 hours of administration. However, after 14 days of administration, AZA only increased urine outputs in wild-type mice, but could not exert a diuretic role in AQP1^−/−^ mice contrarily. AQP1 deficiency blunted the diuretic effect of AZA on day 14.

### AZA decreased AQP1 protein content not by reducing synthesis but by promoting degradation

**Figure 3:**
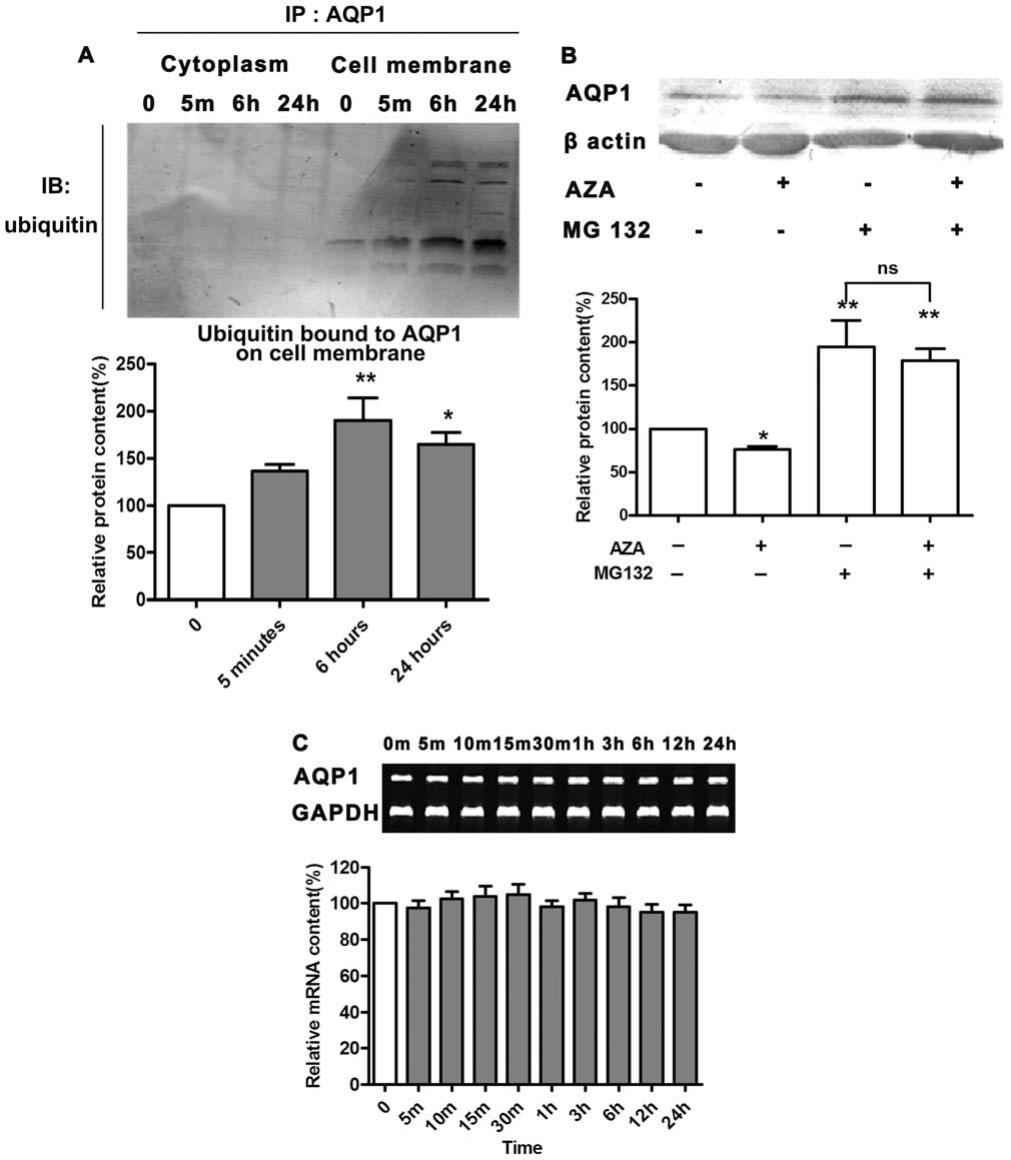
AZA decreased AQP1protein content by promoting AQP1 degradation. **A**, The time-course effect of AZA on ubiquitinated AQP1 protein expression. HK-2 cells were treated with 3×10^−6^ mol/L AZA for 5min, 6 h and 24 h. Control cells were treated with vehicle. Cell membrane and cytoplasm were separated. After immunoprecipitation with AQP1 antibody, cell lysates was determined with ubiquitin antibodies. Each lane was loaded with 60 μg of total protein. The representative blotting image of ubiquitin is shown (up panel). IP and IB are short for immunoprecipitation and immunoblot, respectively. Statistical data is shown (down panel). Results are expressed as a percentage of the control. Values are presented as means±S.E.M. *p<0.05, **p<0.01 compared to control. **B**, Effect of AZA on AQP1 expression in the presence of MG132. HK-2 cells were pretreated with MG132 (10 μM) or vehicle for 8 h, then incubated with AZA for 24 hours. A representative blotting image and summary data are shown. Values are the means±S.E.M. *p<0.05, **p<0.01 compared to control. **C**, Effects of AZA on AQP1 mRNA in HK-2 cells. Cells were incubated with 3×10^−6^ mol/L AZA for a series of times. RT-PCR analysis was performed for the detection of AQP1 and glyceraldehyde-3-phosphate dehydrogenase (GAPDH) mRNAs. Values are presented as means±S.E.M.

Above results told us that AZA exerted diuretic effect also by decreasing AQP1 protein expression besides of CAs inhibition, and when AQP1 was absent in AQP1 null mice urine-increasing effect of AZA was abolished. However, the underlying mechanism how AZA decreased AQP1 expression is still obscure. To elucidate the possible mechanism, we then utilized human kidney-2 (HK-2) cells as an *in vitro* tool to discover how this inhibition occurred, and in pre-experiments, we found AZA incubation for 24 hours could significantly reduce AQP1 protein level in HK-2 cells (data not shown). At first, we focused on whether AZA could affect AQP1 mRNA level because transcriptional regulation is a fundamental manner of protein expression change. We used RT-PCR amplification of AQP1 mRNA prepared from HK-2 cells to examine AQP1 mRNA content. Unfortunately, it did not change from 5 minutes to 24 hours in a medium contained 3×10^−6^ mol/L AZA (Fig 3C).

Besides of transcriptional regulation, the other mechanism about AQP1 expression change is ubiquitin-proteasome mediated degradation [15]. Given that AZA did not change mRNA level of AQP1, next we measured degradation of AQP1. HK-2 cells were incubated in 3×10^−6^ mol/L AZA, and then cell membrane and cytoplasm were separated. Fig 3A showed that after immunoprecipitation by AQP1 primary antibody and then immunoblotted by uibiquitin antibody, AQP1 ubiquitination only occurred on cell membrane and the ubiquitination significantly increased after AZA administration and its peak appeared after drug incubation for 6 hours. It was suggested that AZA might decrease AQP1 expression by enhancing uibiquitin mediated degradation. To confirm the role of ubiquitin-proteasome in inhibitory effect of AZA on AQP1, we found proteasome inhibitor MG132 could prevent AQP1 protein from being decreased after AZA was added (Fig 3B).

### MHC bound AQP1 and mediated AQP1 translocation onto cell membrane

It has been shown that AQP1 degradation through ubiquitin-proteasome system mediated inhibitory effect of AZA, and additionally we noticed that AQP1 ubiquitination only occurred on cell membrane. Considering that previous work has proved translocation between cytoplasm and membrane was an important way of AQP1 content regulation [16] and in our study AQP1 ubiquitination singly happened on cell membrane, we then hypothesized that AZA may firstly promote AQP1 translocation onto cell membrane before inducing ubiquitination of AQP1. Consistently with this hypothesis, we demonstrated AQP1 in the cytoplasm decreased dramatically after AZA treatment, but by contrast, AQP1 on the cell membrane increased after administration and subsequently was reduced in different time-points of AZA administration (Fig S3).

**Figure 4:**
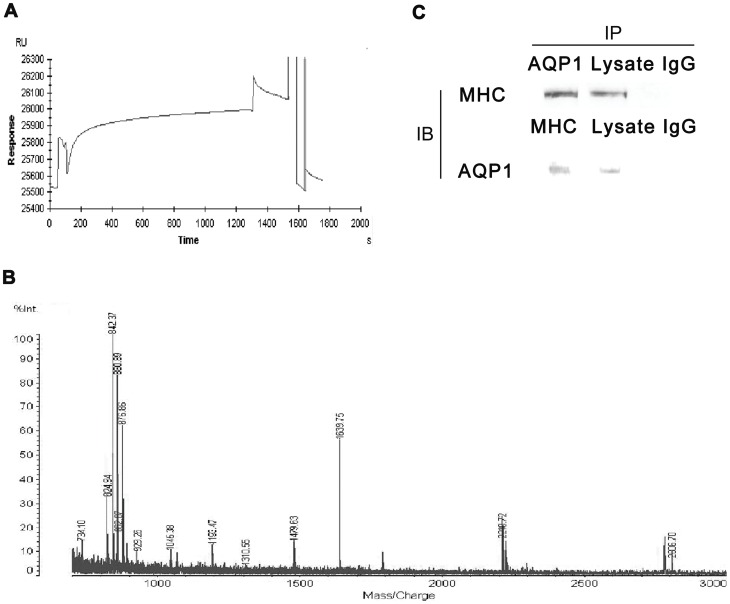
AQP1 and MHC interaction. **A**, Immobilization of AQP1 on CM5 sensor chip. Firstly, Carboxyl groups react with NHS/EDC mixture and reactive NHS esters are formed. Secondly, AQP1 solution is injected and the coupling reaction proceeds. The AQP1 protein purified from rat red blood cells was immobilized onto the CM5 chip. Thirdly, Remaining reactive NHS ester sites are blocked with ethanolamine hydrochloride (pH 8.5). The final AQP1 surface is ready. At last, Sensorgram of AQP1binding protein capture on and recovery from AQP1 immobilized CM5 chip. The binding protein was isolated from rat kidney extract by surface plasmon resonance according REVOVERY program. **B**, The recovered protein that bound to AQP1 was measured by MALDI-TOF analysis. Peptide signals corresponding to the fragments of myosin heavy chain are numbered. **C**, co-immunoprecipitation analysis. 0.5 mg/ml of total extracts were immunoprecipited with 2 µg/ml of anti-AQP1 or 2 µg/ml anti-MHC primary antibody. The samples were then loaded onto SDS-PAGE gel and transferred onto PVDF sheet. The latter was incubated with anti-MHC (1∶1000) or anti-AQP1 (1∶1000) overnight. Protein A-Agarose pre-blocked with 10% of BSA and then incubated with the lysate with IgG was used as a negative control (IgG). IP and IB are short for immunoprecipitation and immunoblot, respectively.

**Figure 5:**
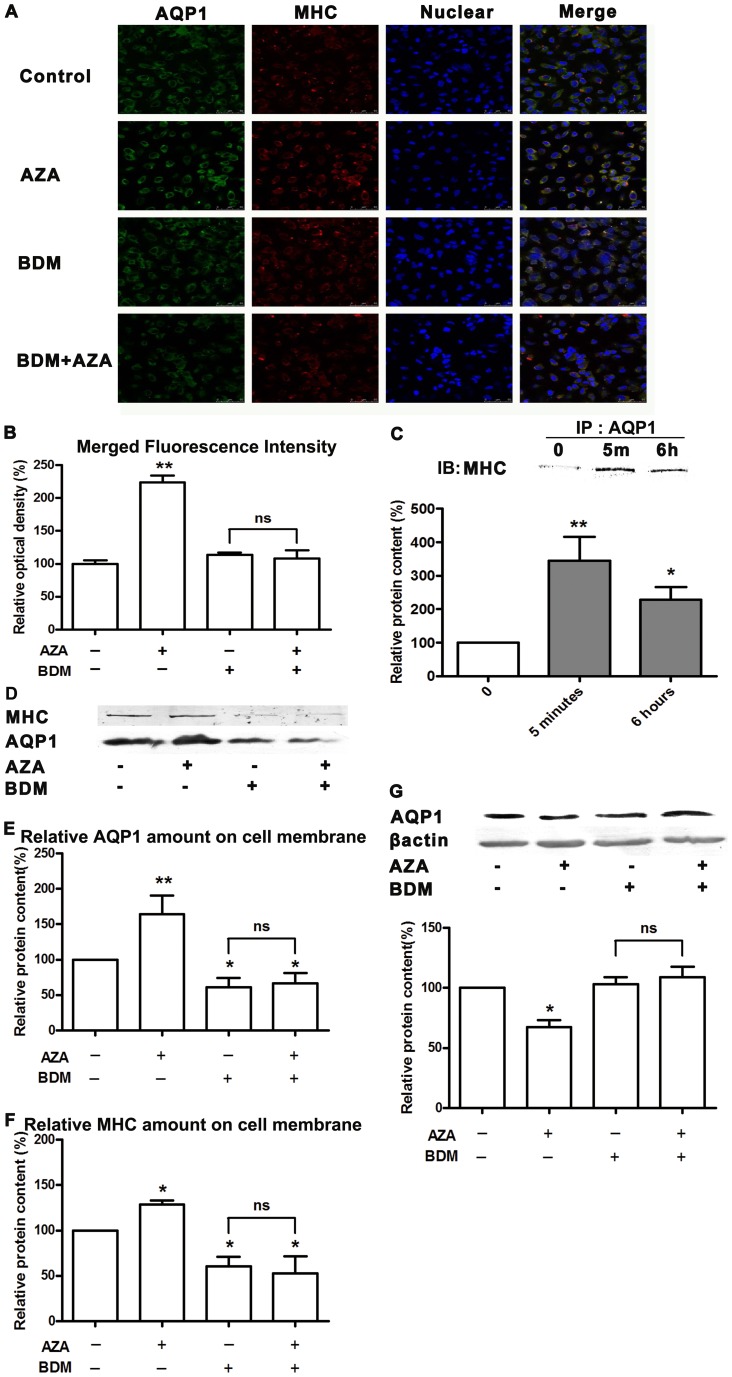
AZA promoted the interaction between AQP1 and MHC. **A**, The confocal laser scanning microscopy images of MHC and AQP1 immunofluorescence in HK-2 cells. HK-2 cells were grown with 3×10^−6^mol/L AZA, 1×10^−2^mol/L BDM or both of them (BDM+AZA) for 6 h. Control cells were treated with vehicle (Control). Blue fluorescent indicates nuclear stained by Hoechst 33342 and the green fluorescent shows the AQP1 staining and the red fluorescent shows the MHC staining. **B**, The yellow stain in merged pictures from Fig. 5A was analyzed by IPP (Image-Pro Plus) software. Results are expressed as a percentage of the control. Values are shown as means±S.E.M. *p<0.05 compared to Control. **C**, The time-course effect of AZA on AQP1 and MHC interaction. HK-2 cells were incubated with 3×10^−6^mol/L AZA for 5min to 24 h. Total extracts were immunoprecipitated with 2 µg/ml of anti-AQP1 antibody. The samples were then loaded onto SDS-PAGE gel and immunoblotted on PVDF sheet. The latter was incubated with anti- MHC (1∶1000) overnight. The representative blotting image of MHC is shown (up panel) and summary data is shown (down panel). Results are expressed as a percentage of the control. Values are presented as the means±S.E.M. *p<0.05, **p<0.01 compared to Control. **D**, The effect of AZA on AQP1 and MHC expression in the presence of myosin inhibitor BDM. HK-2 cells were treated with vehicle, 3×10^−6^mol/L AZA, 1×10^−2^mol/L BDM or both of them for 6 h. Cell membrane was separated and determined by western blot analysis with AQP1 and MHC antibodies. The representative blot of AQP1 and MHC is shown. **E and F**, Summary data from immunoblotting results. Results are expressed as a percentage of the control. Values are shown as means±S.E.M. * *p*<0.05, **p<0.01 compared to Control. **G**, BDM reversed AQP1 reduction induced by AZA. HK-2 cells were treated with vehicle, 3×10^−6^mol/L AZA, 1×10^−2^mol/L BDM or both of them for 24 h. Cell lysates were detected with AQP1 antibodies. The representative blotting image of AQP1 is shown. Statistical data is shown. Results are expressed as a percentage of the control. Values are shown as means±S.E.M. * *p*<0.05 compared to Control. Ns, non-significant.

Above results led us to a further question: How AZA promoted AQP1 translocation onto cell membrane? Since it has been reported microtubules belonging to motor proteins in cells may participate in translocation of AQP1 [17], To discover whether AZA affected AQP1 trafficking onto the membrane, we firstly focused on uncovering which exact motor protein in cytoskeleton family may mediate trafficking of AQP1 by putative mutual binding. To detect AQP1-binding proteins, we established a Surface Plasmon Resonance (SPR)-based method, which is usually utilized to discover molecules interaction. The CM5 chip was activated using NHS/EDC mixture, immobilized with the purified AQP1 and blocked by ethanolamine hydrochloride (Fig 4A). Response Units (RU) indicated the optical changes at the detector surface as proteins binding with or release from AQP1 on the sensor chip and represented the progress of association and dissociation. As shown in Fig 4A, about 500RU analyte was obtained from kidney extract during the RECOVERY program. The recovery experiment was replicated for four times. To identify the AQP1 binding proteins, the recovered content from the kidney extract were detected by Matrix-Assisted Laser Desorption/Ionization-Time of Flight/Mass Spectrometry (MALDI-TOF/MS). The resulting peptides from the analyte (Fig 4B) were mostly correspondent to the common sequence of myosin heavy chain (MHC), member of motor proteins family. Furthermore, we utilized the immunoprecipitation to validate MHC binding to AQP1, and as Fig 4C shown, MHC interacted with AQP1 in rat kidney lysate. In HK-2 cells, immunofluorescence images (Fig 5A and Fig 5B) showed that AQP1 and MHC also co-localized in HK-2 cells.

### AZA promoted AQP1 translocation onto cell membrane through enhancing interaction between AQP1 and MHC

Next we examined whether AZA could affect the co-localization of AQP1 and MHC in HK-2 cells, we concluded that the increased merged fluorescence intensity indicated the co-localization was enhanced when cells were treated with AZA (Fig 5A and Fig 5B). However, if BDM, a MHC inhibitor, was present, AZA did not promote the co-localization (Fig 5A and Fig 5B). Immunoprecipitation result confirmed that AZA promoted AQP1 and MHC interaction, showing that AZA increased the interaction of AQP1 and MHC from 5min to 6 hours (Fig 5C).

Above results suggested that AZA enhanced AQP1 and MHC interaction, next we tried to verify AZA promote AQP1 translocation onto cell membrane in this process. Then we prepared cell membrane lysates and examined the AQP1 (Fig 5D and Fig 5E) and MHC (Fig 5D and Fig 5F) content by western blot analysis. AZA increased both AQP1 and MHC content in cell membrane at 6 h. These results supported our hypothesis that AZA could promote AQP1 translocation onto cell membrane before inducing AQP1 ubiquitination and degradation, in a MHC-dependent manner. So when cells were incubated MHC inhibitor BDM at 1×10^−2^ mol/L, AQP1 translocation was abolished (Fig. 5D and 5E) and consequently down-regulating effect on AQP1 by AZA was cancelled (Fig. 5G).

### Activation of ERK/MLCK/MLC pathway by AZA possibly participated in the AQP1 translocation and reduction

**Figure 6:**
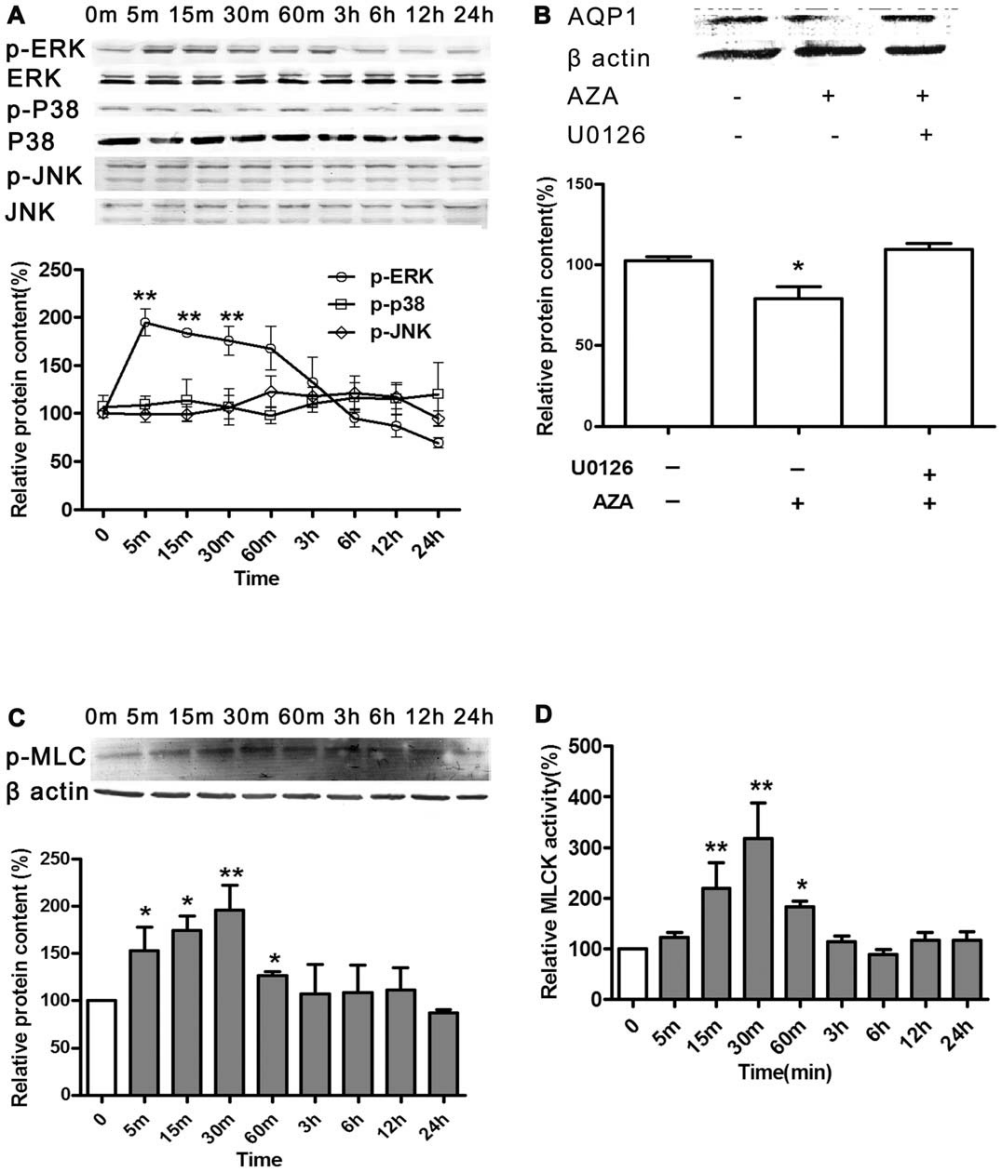
Signaling pathway participated in the AQP1translocation and degradation induced by AZA. **A**, The effects of AZA on the activation of MAPK signaling pathway in HK-2 cells. For the MAPK immunoblotting, cells were incubated with 3×10^−6^ mol/L AZA for a series of times. Cells were harvested. Immunoblot analysis was performed by using antibodies to p-ERK, ERK, p-p38 kinase, p38 kinase, p-JNK, JNK, respectively. Representative blots and summary data are shown. Data is expressed as means±S.E.M. **p<0.01 compared to Control. **B**, The effect of AZA on AQP1 expression in the presence of ERK inhibitor U0126. After pretreated with U0126 (2 mol/L) or vehicle for 30 min, cells were incubated with AZA for 24 hours. Values are as means±S.E.M. * *p*<0.05 compared to Control. **C**, The effect of AZA on the activation of MLC in HK-2 cells. For p-MLC immunoblotting, cells were incubated with 3×10^−6^ mol/L AZA for a series of times. Cells were harvested. Immunoblot analysis was performed by using antibodies to p-MLC and summary data is shown here. Values are presented as means±S.E.M. * *p*<0.05, **p<0.01 compared to Control. **D**, The effect of AZA on MLCK activity. Cells were incubated with 3×10^−6^ mol/L AZA for a series of times. Cells were harvested and MLCK activity was analyzed by ELISA assay. Summary data is shown. Values are expressed as means±S.E.M. * *p*<0.05, **p<0.01 compared to Control.

Lastly we tested which signaling pathway mediated translocation promoting role of AZA on AQP1. It has been proposed that mitogen-activated protein kinase (MAPK) signaling pathway plays an important role in AQP1 content regulation [18], [19], and also downstream of external-signal regulated kinase (ERK)-MAPK myosin light chain kinase (MLCK), which phosphorylates myosin light chain (MLC) is an crucial modulator of MHC activity [20]. After HK-2 cells being treated with AZA in a concentration of 3×10^−6^ mol/L at different time points, MAPK family members (ERK, p38, c-Jun N-terminal kinase (JNK)) and MLC as well as their activate forms, which may take part in regulation of AQP1 and MHC, were examined by western blot analysis. The results (Fig 6A) showed that phosphorylation levels of JNK and p38 did not change significantly. But AZA induced activation of ERK in a time-dependent manner. The peak of phosphorylation of ERK was observed to occur after 5 minutes of AZA treatment, and then weakened to control level post-treatment. MLC were phosphorylated from 5 minutes to 30 minutes (Fig 6C). The activity of MLC upstream MLCK was tested by ELISA assay (Fig 6D). MLCK activity began to increase in 5min, after 30min administration it reached peak, then after 3 hours it returned to original level.

ERK/MLCK/MLC pathway activation orderly after AZA treatment gave us the clue that this signaling pathway eventually activated MHC to interact with AQP1, then promoting translocation onto cell membrane and reduction by degradation. To validate the role of this ERK-dependent pathway, we used U0126 specifically inhibiting ERK and found it prevented AQP1 from being increased on cell membrane after AZA incubation (data not shown) and finally reversed the AQP1 reduction induced by AZA (Fig 6B). Moreover, MLCK inhibitor wortmannin pre-incubation also prevented AQP1 from being brought down by AZA, suggesting a possible role of MLCK activation in down-regulating effect of AZA (Fig S3).

## Discussion

Aquaporins are a family of homologous intrinsic membrane proteins that are responsible for the water permeability of membranes in a variety of cell types [14]. To date, there are at least 8 types of aquaporins (AQP1, 2, 3, 4, 6, 7, 8 and 11) known to express in the kidney [21]. Among those family members, AQP1 is abundant in the apical and basolateral membranes of epithelial cells of the proximal tubule, consistent with a role for transcellular water re-absorption in the proximal nephron [22]. The critical role of AQP1 in urinary concentration was confirmed in transgenic knockout mice lacking the AQP1 gene [23]. AQP1-deficient mice display diuresis and are unable to concentrate urine even in the deprivation of water, a condition whereby they rapidly become dehydrated. Subsequent studies have demonstrated that the osmotic water permeability of isolated perfused proximal tubules from AQP1 null mice possessed only 20% the capacity of fluid re-absorption compared to wild type mice [24]. This finding of impaired fluid re-absorption in proximal tubules of AQP1 knockout mice implies that AQP1 might be a unique target of inhibitors that may be capable of inducing diuresis [25]. Thus, discovering of AQP1 inhibitor has drawn intensive attention of pharmacological researchers. Till now, several ions or small molecules have been considered as potential AQP1 inhibitors, including Hg ^2+^ [26], TEA^+^ [27], AZA [28] and AqB013 [29]. Among these inhibitors, AZA was firstly indentified by our lab to act as AQP1 inhibitor [11], [28], and after that several groups have confirmed this finding [12], [13], [30]–[32]. Also, based on structure of AZA and bumetanide, novel synthesized compound was discovered to be a putative inhibitor of AQP1 [29], [33]. However, other reports demonstrated AZA could not inhibit water transportation function of AQP1 by different assays [34], [35]. We considered the controversial results might be caused by various assay methods and different incubation time of AZA.

In this present study, we aimed to confirm the previous finding that AZA modulates AQP1 and further elucidate its underlying mechanism. Firstly we found that increase of urine volume induced by AZA was not only explained by CAs activity inhibition but also AQP1 reduction. Both of them were contributed to AZA's diuretic effect. The initial diuretic effect was induced by CAs activity inhibition. But CAs activity gradually recovered within the administration time, and we noticed that CAs activity even returned to normal level on day 14. The urine volume result from AQP1^−/−^ mice validated the critical role of AQP1 in the long-term diuretic process. As is reported, AQP1 deficiency produced remarkable diuretic effect before administration. In AQP1^−/−^ mice, because of absence of AQP1, AZA's diuretic effect was abolished when CAs activity totally recovered on day 14 after dosing. By contrast, for wild type mice, when AQP1 was present, AZA produced notable diuretic effect on day 14 after administration despite of recovering of CAs activity. On day 14, if AQP1 was not the only or main target of AZA, AZA would increase urine volume in AQP1 knockout mice through other mechanisms despite absence of AQP1. However, on Day 14 we demonstrated AZA could not increase urine volume in knockout mice compared to wild type mice (Fig 1B).Thus, AQP1 reduction played a major role in diuretic effect of AZA when CAs activity recovered.

When we applied NaHCO_3_ to the rats together with AZA treatment to modify metabolic acidosis, then changes of AQP1, CAII and CAIV protein contents and CAs activity was observed. When NaHCO_3_ was present, CAII and CAIV expressions were not induced by AZA and CAs activity was still at a low level on day 14. AZA has been reported to be rapidly tolerated when clinically used, and one of the important reasons is its impact on disturbing acid/base balance by inducing acidosis. It has been demonstrated that acidosis circumstance induced increase of carbonic anhydrase mRNA expression and activity recovery [36]. Thus, metabolic acidosis weakens the diuretic effect of AZA by restoring CAs activity. Consistently, in our present study, we found single use of AZA caused significant CAs proteins induction and CAs activity recovery (Fig 2), but when combined with NaHCO_3_ to eliminate acidosis condition, CAs were not induced by AZA any longer (Fig 2).

However, under combination of AZA and NaHCO_3_, AQP1 was still suppressed. The urine volume in NaHCO_3_ (+) group was significantly higher than that in NaHCO_3_ (−) group on day 14 of administration, because now the diuretic effect come from adding of both AQP1 and CAs inhibition, which indicates that inhibiting AQP1 or CAs by AZA are two independent events in kidney both leading to diuresis. To further exclude the impact of NaHCO_3_ in our present study, we added a group of rats receiving only 30 mg/kg/day of NaHCO_3_, and urine volume, CAs activity and expression, as well as AQP1 protein expression were measured. We found single use of NaHCO_3_ could slightly elevate rats' urine volume compared with control group, but only on Day 7 there was statistical significance (Fig S1A). This could explain why urine volume was different between single use of AZA and combination of AZA and NaHCO_3_ (Fig. 1A) because NaHCO_3_ itself had a mild diuretic effect, which is consistent with previous reports [37]. In addition, to elucidate the impact of NaHCO_3_ alone on CAs and AQP1, we demonstrated that single use of NaHCO_3_ could not significantly influence either activity (Fig S1B) or expression of CAs (Fig S1C and S1D), and moreover, AQP1 expression was not changed due to single NaHCO_3_ administration either (Fig S1C and S1D). Thus, we concluded that NaHCO_3_ alone only exerted a slight diuretic effect and had no impact on CAs and AQP1 content. Above all, we speculate that the rapid diuresis caused by AZA is due to the inhibition of CAs activity. When CAs activity recovers, a novel diuretic mechanism of long-term treatment of AZA is suppression of AQP1 protein expression.

Now we conclude that AZA can exert diuretic effect by down-regulating AQP1, next we focused on explaining possible inhibitory mechanism. Our present study for the first time demonstrates that AZA can influence AQP1 translocation in a myosin heavy chain-dependent manner via activation of ERK pathway and its downstream signal cascade. Translocation or trafficking is an important way for aquaporins post-transcriptional regulation, among which trafficking of AQP2 has been firstly mentioned and already studied a lot. Now it is well known that vasopressin can induce translocation of AQP2 into the apical plasma membrane in renal collecting duct cells [38]. Similar to AQP2, translocation of AQP1 has also been the target of intensive investigation. AQP1 translocation between cell membrane and cytoplasm has been reported in many different cell types, including transfected Chinese hamster ovary cells [39], cholangiocytes [40], [41], peritoneal mesothelial cells [42], astrocytes [16], transfected HEK293 cells [17] and so on. It was firstly identified that the expressed CHIP28k protein is a selective water channel that is functional in endocytic vesicles and the cell plasma membrane [39]. Another *in vitro* study has implicated the vesicular translocation of AQP1 water channels to the plasma membrane, a mechanism that appears to be essential for secretin-induced ductal bile secretion in cholangiocytes and suggested that AQP1 can be regulated by membrane trafficking [40]. Furthermore, results from *in vivo* study indicated that secretin induces the insertion of AQP1 exclusively into the secretory pole, a process that depends on microtubules [17], [41]. Here we show AZA is a stimulating factor for AQP1 translocation for the first time. In our study, we found that AQP1 in the cytoplasm decreases, by contrast, AQP1 on the cell membrane increases after administration (Fig. S3).

Consistently with previous reports, in our present study, we have found out that translocation of AQP1 needs interaction with motor proteins, and also identified the exact binding protein is myosin heavy chain (MHC) since SPR has been considered as a powerful and reliable tool to discover novel protein interaction [43] and also our immunoprecipitation and immunofluorescence results both confirmed the binding between AQP1 and MHC. Therefore, we hypothesized that MHC may provide drive for trafficking of AQP1. Very likely, it has been elucidated that kinesin and dynein-also members of motor protein family-are present in both cholangiocyte lysates and in isolated AQP1-containing vesicles, which play important roles in translocation of AQP1 as motor proteins [44]. Interestingly, as to AQP2, AQP2-binding proteins that directly regulate its trafficking have been uncovered, including cytoskeleton protein actin and several motor proteins including myosin IC, non-muscle myosin IIA and IIB, myosin VI, myosin IXB[45], [46], and especially similar to our present study myosin heavy chain non-muscle type A[47]. These work proved our finding of motor protein participating in translocation of aquaporins, and furthermore we provide new evidences to show MHC acts as an important component in trafficking of AQP1. By using BDM, a MHC inhibitor, we found out that BDM can diminish the interaction between MHC and AQP1, and also BDM abolishes the translocation of AQP1 onto the membrane. However, despite of above data concerning MHC, we still could not consider MHC as the only mediator of AQP1 translocation, and future work to examine MHC and other possible cytoskeleton proteins expression in AQP1-containing vesicles is still needed.

Action of myosin heavy chain needs regulation and binding of myosin light chain, which can be phosphorylated by myosin light chain kinase. In our experiments, we found that upstream of MLCK-signal molecule ERK-has been activated by AZA. Therefore, we propose that AZA induces phosphorylation of MLCK via activation of ERK pathway, and then promotes interaction between MHC and AQP1 to increase the translocation of AQP1. As predicted, ERK pathway inhibitor U0126 can reverse the effect of AZA on AQP1. In addition, like U0126, MLCK non-specific wortmannin also abolished down-regulating effect of AZA upon AQP1. Besides, U0126 or wortmannin alone could not affect expression of AQP1 in our present study (data not shown). Therefore, we consider ERK-MLCK pathway as an important mediator in suppression effect of AZA upon AQP1. However, there is one limitation that that wortmannin is a non-specific inhibitor of MLCK [48], thus, and in future work, we need specific inhibitor of MLCK to better validate its role.

A lot of signaling pathways have been proved to take part in trafficking and content regulation of aquaporins. Due to AQP2 translocation, cAMP-protein kinase A (PKA) pathway has a crucial role; however, other signaling cascades also induce AQP2 translocation, such as phosphoinositide 3-kinase (PI3K) pathway [49]. Moreover, translocation of AQP1 has also been related to PKA pathway [42]. In our *in vitro* study, we have examined the level of cAMP after administration of AZA, however, the change of cAMP level is not consistent with trafficking of AQP1 (data not shown). In another study, researchers have demonstrated that ultraviolet radiation (UVB) and H_2_O_2_ induced AQP1 down-regulation which was mediated by ERK activation, however, the mechanism was not mentioned in this article [50]. Our results may explain the effect of ERK pathway in translocation and down-regulation of AQP1.There are still distinct findings to identify signaling pathway associated with trafficking of AQP1, such as one recent study reported that AQP1 trafficking was mediated by the tonicity of the cell environment in a specific PKC- and Ca^2+^-dependent manner [16], [17]. Therefore, signaling cascades for translocation of AQP1 still need further examination.

Eventually we focused on the fate of AQP1 which has been transported onto cell membrane after administration of AZA. Previous work of our lab has proved that AZA binds directly to AQP1 and inhibits water permeability [10], [11]. Therefore, we hypothesized that AQP1 bound by AZA may be recognized by ubiquitin-proteasome system to initiate degradation of AQP1 because it has been indentified that AQP1 degradation is dependent on ubiquitin-proteasome pathway [15]. Our study showed that after translocation onto the membrane, AQP1 is linked and signed by ubiquitins because we find an increase of ubiquitin bound to AQP1 after administration of AZA, and also ubiquitination appears only on the cell membrane. Therefore, in our study, giving proteasome inhibitor MG132 can reverse the inhibitory effect of AZA on AQP1. Now we can summarize that although AZA promotes translocation of AQP1 onto the membrane, the protein is finally degradated through ubiquitin-proteasome system. Given the results that AZA did not influence mRNA expression of AQP1 by RT-PCR examination, we can ascribe the reduction of AQP1 after administration of AZA for promotion of translocation and degradation. That is to say, AQP1 protein synthesis does not change, however its degradation levels up. This may account for the down-regulating effect of AZA on AQP1 protein.

In conclusion, the present study provides evidence that AZA has different diuretic mechanisms at different time points. At beginning, AZA inhibits carbonate anhydrases activity. Then CAs activity recovers and the diuretic mechanism of AZA is caused by reducing AQP1 protein expression. The reduction of AQP1 is due to increase of translocation onto the cell membrane dependent on ERK/MLCK pathway and interaction with MHC, and then degradation through uibiquitin-proteasome system. Our study implicates an innovative mechanism for action of diuretics AZA and also provides a novel sight for diuretic agent discovery.

## Materials and Methods

### Agents

AZA, MG132, U0126, Wortmannin and BDM were from Sigma–Aldrich Corporation (Sigma Chemical Co., St. Louis, MO). Biacore3000 CM5 series sensor chips were purchased from Biacore AB (Piscataway, NJ). Other biochemical reagents were from Beijing Chemical Plant (Beijing, China).

### Ethics Statement

This study was performed in strict accordance with the recommendations in the Guide for the Care and Use of Laboratory Animals of China Association for Laboratory Animal Science. All animal care and experimental protocols were approved by the Animal Care Committee of Peking University Health Science Center. All sacrifice was performed under pentobarbitone anesthesia, and every effort was made to minimize suffering.

### Experimental animals and measurement of urine volume

Male rats of Sprague Dawley strain, initially weighting 160 g, were obtained from the Animal Center of Peking University Health Science Center and AQP1^−/−^ and wild-type mice were gifts from Professor Yang Baoxue in Peking University. Rats were divided into 18 groups randomly. Five to seven rats for each group were treated with AZA at 40 mg/kg/day or vehicle for 8 h, 1, 3, 7, 14 days with or without combination with 30 mg/kg/day NaHCO_3_. Rats were maintained on axenic laboratory chow with free access to water. All rats were kept in metabolic mages in a Specific Pathogen Free (SPF)-level laboratory. After administering drugs or vehicle 8 hours, urine volumes of rats were measured and recorded. AQP1^−/−^ and wild-type mice were randomly divided into 3 groups respectively. Six mice for each group were treated with vehicle or AZA at 60 mg/kg/day for 8 h and 14 days. Animals were kept in metabolic mages in a SPF-level laboratory and free access to water. Urine volumes of mice were measured pre-treatment and after administering vehicle or drugs.

### Carbonic anhydrase activity assay

An endpoint colorimetric microtechnique [51] was used to assay carbonic anhydrase activity. The reaction system contained 0.5 mL of buffer (20 mmol/L imidazole, 5 mmol/L Tris, 0.2 mmol/L p-nitrophenol) and 0.5 mL diluted protein sample. One enzyme unit (EU) corresponds to the activity resulting from a reaction time of one-half the time obtained using the same amount of heat-inactivated sample. Formula to calculate carbonic anhydrase activity:
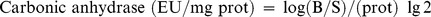
Where B and S are the times measured for paired heat-inactivated enzyme and active sample, respectively; prot is mg of protein contained in the volume of sample used for that particular measurement; lg 2 = 0.301.

### Cell culture

HK-2 cells were purchased from Cell Culture Centre, Institute of Basic Medical Science Chinese Academy of Medical Sciences (Beijing, China). Briefly, HK-2 cells were cultured in DMEM/F12 medium supplemented with antibiotics (penicillin and streptomycin) and 10% fetal bovine serum (Hyclone, Logan, UT) in a humid atmosphere incubator with 5% CO_2_ at 37°C. Cells passaged into 0.5–1×10^5^ cells per 60 mm cell culture dish. The cells were incubated with drugs or vehicle after growing to 70% to 80% confluence, and then cells were harvested and extracted by RIPA lysis buffer containing PMSF (phenylmethylsulfonyl fluoride) and protease inhibitor cocktail (Roche, Basel, Switzerland). Next, lysates were centrifuged at 12,000 g for 10min at 4°C. The supernatants were collected and stored at −80°C. Protein concentration was determined by BCA assay (Thermo Scientific, Rockford, IL).

### Recovery of AQP1 binding protein by Surface Plasmon Resonance (SPR)

AQP1 protein was isolated and purified from the rat red blood cells. The purified AQP1 was injected onto the CM5 chip. Binding activity of AQP1 immobilized on the CM5 chip was assayed using AQP1 antibody at an increasing concentration. After immobilization of AQP1, rat kidney extraction was diluted in five-fold of HBS-EP buffer. The insoluble residue was pelleted by centrifugation and discarded. The supernatant was diluted ten-fold in the same buffer (in the following this preparation is referred to as kidney extract), and 200 µL kidney extract was injected at a flow rate of 20 µL/min. HBS-EP buffer was used as the running buffer in this experiment. The capture and recovery of the AQP1 binding proteins was conducted according to the RECOVERY program encoded in the instrument. The flow system was then washed with 50 mmol/L NaOH and the flow cells were rinsed with 50 mmol/L NH_4_HCO_3_, 2 mmol/L CaCl_2_. The bound proteins were eluted with 2 µL of 0.1% TFA and deposited to a vial in a sample rack for the following analysis.

### Identification of the recovered protein by MALDI-TOF/MS

The masses of the peptides obtained above were analyzed by MALDI-TOF/MS (Matrix-Assisted Laser Desorption/Ionization-Time of Flight/Mass Spectrometry) on an AXIMA-CFR Plus MALDI-TOF mass spectrometer (Shimadzu Biotech). The peptide masses were submitted to the MSDB database via the Mascot database search engine (Matrix Science) for protein identification.

### Immunoprecipitation

Rats were injected with pentobarbitone (100 mg/kg) i.p. to induce anesthesia. The kidney was removed and washed three times in ice cold PBS (pH 7.4), and then cortex portion was homogenized in lysis buffer. HK-2 cells were lysised in the RIPA buffer on ice. The homogenate was then centrifuged at 12,000 g for 10 min at 4°C. The protein concentration of the supernatant was determined using the BCA assay. 0.5 mg/ml protein was added in 1 μg/mL IgG and 20 μl/ml protein A-Agarose (Santa Cruz, SC, CA). After centrifugation 1,000 g for 5min at 4°C, the supernatant was added in 10 μl/ml AQP1 or MHC antibody at 4°C for 1 hour. Then 20 μl/ml protein A- Agarose was added to the lysate and mixed at 4°C overnight. After centrifugation 1,000 g for 5min at 4°C, the supernatant was removed. Wash the pellet 4 times and take a part of the protein for protein concentration determination. The others were re-suspended with loading buffer. The sample was boiled for 5min, and then centrifuged at 4°C, at 1,000 g for 5min. Finally, obtained samples were analyzed by Western blot analysis.

### Western blot analysis

Samples (60 μg) were separated by 12% SDS-PAGE. Upon completion of the electrophoresis, the proteins were transferred to PVDF membrane. Non-specific binding sites were blocked by pre-incubating the membrane in 5% skimmed milk in Tris-buffered saline Tween (TBS-T, 20mmol/L Tris, 137 mmol/L NaCl, pH 7.6). After PVDF Membranes were incubated overnight at 4°C with specific antibodies: anti-p-ERK (Tyr204), anti-ERK1/2, anti-p-p38 (Thr180/Tyr182), anti-p38, anti-p-JNK (Thr183/Tyr185), anti-JNK, anti-carbonic anhydrase II, anti-carbonic anhydrase IV, anti-MHC and anti-AQP1 (1∶1000, Santa Cruz, SC, CA) and washed three times with TBS-T buffer, the membranes were incubated with alkaline phosphatase-conjugated goat anti-rabbit or goat anti-mouse IgG (1∶2000, PIERCE, Rockford, IL). Then the western blot bands were scanned, and bands intensity was analyzed by Bio-Rad Quantity One software for quantification.

### RT-PCR

HK-2 cells were harvested and total mRNA was prepared by a commercial available kit (Bioteke, Beijing, China). The RNA was quantified spectrophotometrically, by measuring absorbance at 260 nm (A260), and its purity was determined by the ratio of A260/A280. 5 μg each of total RNA together with the oligo (dT) _18_ and ReverAid^TM^ M-MuLV reverse transcriptase (Fermentas, Life sciences, UK) were used to generate the first strand cDNA mix. The mixture were incubated for 1 hour at 42°C, then the reverse transcriptase was inactivated by heating to 95°C for 5min and the contents were cooled to 4°C before allocating for polymerase chain reaction (PCR). Sequence specific primers for human AQP1 (5′-AGATCAGCATCTTCCGTG-3′ and 5′-AGTTGTGTGTGATCACCG-3′) [52] and for human GAPDH (5′-AACGGATTTGGTCGTATTG**-**3′ and 5′-GCTCCTGGAAGATGGTGAT**-**3′) were employed in the PCR system. Amplification was performed in a thermal cycler (PERKIN ELMER, Gene Amp PCR System 2400) using the following program: initial melt at 94°C for 3 minutes, 55°C for 1minute, 31 cycles at 95°C for 45 seconds, 55°C for 40 seconds, and 72°C for 45 seconds, followed by a final extension at 94°C for 1minute and 60°C for 10 minutes and storage at 4°C. The products were analyzed using 1% agarose gels with DNA ladders.

### Immunofluorescence

HK-2 cells were grown on poly-lysine treated glass slide. Firstly, Wash 3 times with PBS before staining. After fixation by 4% paraformaldehyde at room temperature for 20min, cells were penetrated by 0.5% Triton X-100 at room temperature for 20min. Then wash 3 times with PBS. Next cells were incubated by a normal corresponding serum working solution at room temperature for 30min. Primary antibodies for AQP1 and MHC were incubated at 4°C overnight, subsequently the appropriate secondary antibody was incubated at 37°C for 1 hour. Nuclear was stained by Hoechst 33342 of 1 min. Lastly Wash 3 times with PBS. The images were recorded by confocal microscope (Leica TCS SP5, Japan).

### ELISA

The myosin light chain kinase (MLCK) ELISA kit was purchased from Beijing ZhongHaoShiDai Biotechnology Center (Beijing, China). Briefly, 100ul sample was added into one well. Cover microfilter plates and react at 37°C for 90 minutes. After being washed twice, biotin antibody working solution was added to wells. Then react at 37°C for 60 minutes. After washing 3 times, ABC working solutions were added to wells. Then react at 37°C for 30 minutes. Following washing for 5 times, TMB color working solution was added to wells at 37°C in dark. Then add TMB stop solution to wells. OD values of 450 nm were examined by ELISA Reader (Thermo Scientific, Rockford, IL).

### Analysis and statistics

All results are expressed as means ± S.E.M. For multiple comparisons, the statistical analysis was performed by using one-way ANOVA followed by the Newman-Keuls multiple comparison tests. *P*<0.05 was considered to be statistically significant.

## Supporting Information

Figure S1 **Impact of NaHCO_3_ alone on rats urine volume, carbonic anhydrases activity and expression, as well as aquaporin-1 expression.**
(DOC)

Figure S2 **Effect of MLCK inhibitor wortmannin on AQP1 expression.**
(DOC)

Figure S3 **Time-course effect of acetazolamide on AQP1 protein expression on the cell membrane and cytoplasm.**
(DOC)

Table S1 **Blood pH values in Rats administrated with acetazolamide combined with or without NaHCO_3_.**
(DOC)
