# Supplementary material for: Aquaporin-1 Translocation and Degradation Mediates the Water Transportation Mechanism of Acetazolamide
Source: PLoS One. 2012 Sep 21;7(9):e45976. doi: 10.1371/journal.pone.0045976 (PMC3448731; doi:10.1371/journal.pone.0045976)
Supplement: Figure S1 — Impact of NaHCO3 alone on rats urine volume, carbonic anhydrases activity and expression, as well as aquaporin-1 expression. (DOC) [file pone.0045976.s001.doc]

**Supporting Information**

**Figure S1** Impact of NaHCO_3_ alone on rats urine volume, carbonic anhydrases activity and expression, as well as aquaporin-1 expression.

**Figure S2** Effect of MLCK inhibitor wortmannin on AQP1 expression.

**Figure S3** Time-course effect of acetazolamide on AQP1 protein expression on the cell membrane and cytoplasm.

**Table S1** Blood pH values in Rats administrated with acetazolamide combined with or without NaHCO_3_.

**
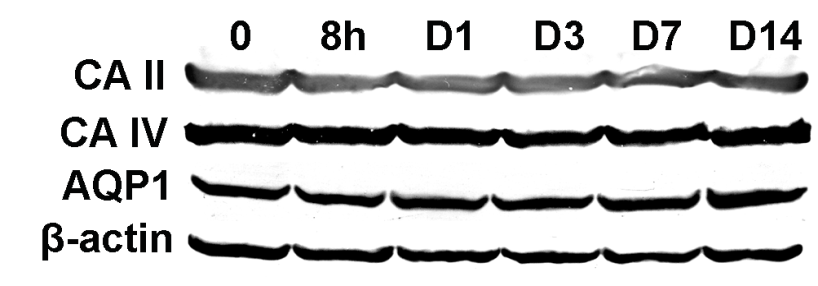
**

**Figure S1**

**A**

**B**

**C**

**D**

Figure S1. Impact of NaHCO_3_ alone on rats urine volume, carbonic anhydrases activity and expression, as well as aquaporin-1 expression. A, The effect of oral administration of NaHCO_3_ (30mg/kg/day) on urine volume in rats. Urine was collected in 8 hours after different administration times (8 hours, 1 day, 3 days, 7 days and 14 days). Values are the means±S.E.M. ******p*<0.05 compared to the control group. B, Time course of total CAs activity in rat kidney cortex. The carbonic anhydrase activity was assayed by endpoint colorimetric microtechnique after the treatment NaHCO_3_ (30mg/kg/day) at the indicated time. Results are expressed as a percentage of the control. Values are shown as means±S.E.M. C, CAs and AQP1 prepared from rat kidney cortex were examined by immunoblotting. Each lane was loaded with 60 μg of total protein from rats at various times after NaHCO_3_ (30 mg/kg/day) treatment. The representative blotting images of CA II, CA IV and AQP1 are shown with β-actin as an internal control. D, Statistical data are shown for CAII (left panel), CAIV (middle panel) and AQP1 (right panel). Results are expressed as a percentage of the control. Values are the means±S.E.M.
