# Supplementary material for: Aquaporin-1 Translocation and Degradation Mediates the Water Transportation Mechanism of Acetazolamide
Source: PLoS One. 2012 Sep 21;7(9):e45976. doi: 10.1371/journal.pone.0045976 (PMC3448731; doi:10.1371/journal.pone.0045976)
Supplement: Figure S2 — Effect of MLCK inhibitor wortmannin on AQP1 expression. (DOC) [file pone.0045976.s002.doc]

**Figure S2**

**
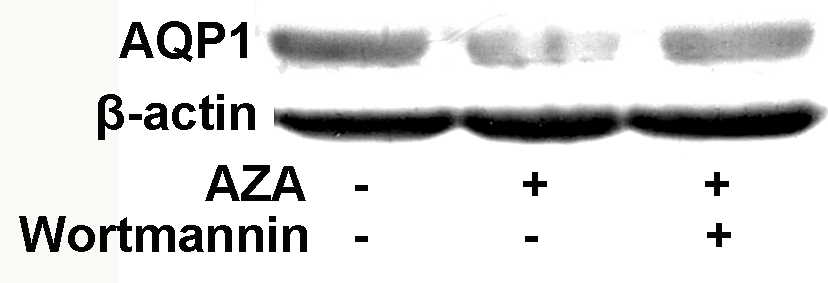
**

**A**

Figure S2 Effect of MLCK inhibitor wortmannin on AQP1 expression. A, The effect of AZA on AQP1 expression in the presence of MLCK inhibitor wortmannin. After pretreated with wortmannin (1 μmol/L) or vehicle for 30 min, cells were incubated with AZA for 24 hours. AQP1 protein expression was measured by immunoblotting. Representative blotting image is shown here (up panel), and also statistical data is presented (down panel). Values are the means±S.E.M. ********p*<0.001 compared to Control.
