# Supplementary material for: Aquaporin-1 Translocation and Degradation Mediates the Water Transportation Mechanism of Acetazolamide
Source: PLoS One. 2012 Sep 21;7(9):e45976. doi: 10.1371/journal.pone.0045976 (PMC3448731; doi:10.1371/journal.pone.0045976)
Supplement: Figure S3 — Time-course effect of acetazolamide on AQP1 protein expression on the cell membrane and cytoplasm. (DOC) [file pone.0045976.s003.doc]

**Figure S3**

**A**

**B**


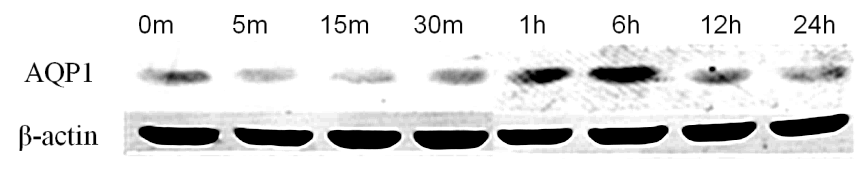

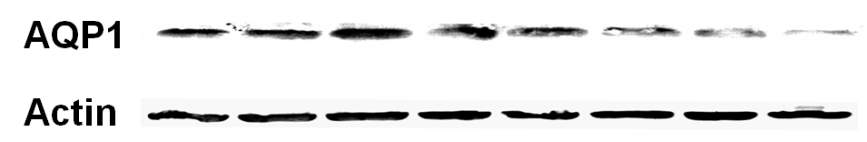

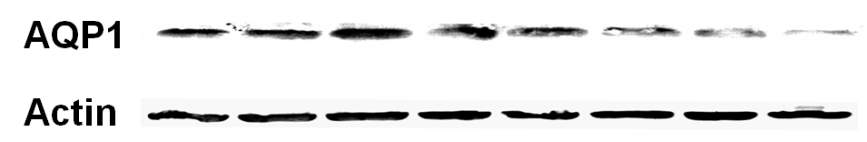


**AQP1**

**0 1h 6h 12h 24h**

**0 1h 6h 12h 24h**

Figure S3. Time-course effect of acetazolamide on AQP1 protein expression on the cell membrane and cytoplasm. A, Time-course effect of acetazolamide on AQP1 protein expression on the cell membrane. HK-2 cells were treated with 3×10^-6^mol/L acetazolamide for 0 to 24h. Control cells were treated with vehicle. Cell membrane and cytoplasm were separated. Cell lysates of membrane were determined with AQP1 antibodies. Each lane was loaded with 60 μg of total protein. The representative blotting image of AQP1 is shown (up panel). Summary data is shown (down panel). Results are expressed as a percentage of the control. Values are the means±S.E.M. *****p<0.05, ******p<0.01 compared to Control. B, Time-course effect of acetazolamide on AQP1 protein expression in cytoplasm. HK-2 cells were incubated with 3×10^-6^mol/L acetazolamide for 0 to 24h. Control cells were treated with vehicle. Cell membrane and cytoplasm were separated. Cell lysates of cytoplasm were determined with AQP1 antibodies. Each lane was loaded with 60 μg of total protein. The representative blot of AQP1 is shown. Statistical data is shown. Results are expressed as a percentage of the control. Values are presented as means±S.E.M. *****p<0.05, ******p<0.01. *******p<0.001 compared to Control.
