# Supplementary material for: Aquaporin-1 Translocation and Degradation Mediates the Water Transportation Mechanism of Acetazolamide
Source: PLoS One. 2012 Sep 21;7(9):e45976. doi: 10.1371/journal.pone.0045976 (PMC3448731; doi:10.1371/journal.pone.0045976)
Supplement: Table S1 — Blood pH values in Rats administrated with acetazolamide combined with or without NaHCO3. (DOC) [file pone.0045976.s004.doc]

**Table S1**

| **Administration times** | **AZA** | **AZA+NaHCO_3_** |
| --- | --- | --- |
| **0** | 7.38±0.04 (n=5) | 7.38±0.04 (n=5) |
| **8h** | 7.20±0.06 (n=7)* | 7.24±0.03 (n=7)* |
| **1d** | 7.21±0.08 (n=10)* | 7.30±0.07 (n=5)# |
| **3d** | 7.25±0.05 (n=8)* | 7.39±0.06 (n=7)# |
| **7d** | 7.25±0.03 (n=7)* | 7.38±0.04 (n=11)# |
| **14d** | 7.24±0.06 (n=7)* | 7.40±0.04 (n=11)# |

Table S1 Blood pH values in Rats administrated with acetazolamide combined with or without NaHCO_3_. Rats were treated with acetazolamide (40 mg·kg^-1^·day^-1^) or acetazolamide (40 mg·kg^-1^·day^-1^) and NaHCO_3_ (30 mg·kg^-1^·day^-1^) for 8 hours (8h), 1 day (1d), 3 days (3d), 7 days (7d) and 14 days (14d). Blood pH value was measured and analyzed. (n) Indicates the number of animals used. Values are shown as means±S.E.M. *****p<0.05 compared to Control. #p<0.05 compared to AZA.
